# Artificial Intelligence Predicts Fitzpatrick Skin Type, Pigmentation, Redness, and Wrinkle Severity From Color Photographs of the Face

**DOI:** 10.1111/jocd.70050

**Published:** 2025-03-26

**Authors:** Rachel L. Draelos, Chelsea E. Kesty, Katarina R. Kesty

**Affiliations:** ^1^ Vismedica AI, LLC Durham North Carolina USA; ^2^ St. Petersburg Skin and Laser St. Petersburg Florida USA; ^3^ Kesty AI St. Petersburg Florida USA

**Keywords:** artificial intelligence, computer vision, Fitzpatrick skin type, hyperpigmentation, laser rejuvenation, laser resurfacing, machine learning, redness, wrinkles

## Abstract

**Background:**

Due to high patient demand, increasing numbers of non‐dermatologists are performing skin assessments and carrying out laser interventions in medical spas, leading to inferior outcomes and higher complications. A machine learning tool that automatically analyzes patient skin has the potential to aid non‐dermatologists.

**Aims:**

To develop a high‐performing machine learning model that predicts Fitzpatrick skin type, hyperpigmentation, redness, and wrinkle severity simultaneously.

**Methods:**

We developed the SkinAnalysis dataset of 3662 images, labeled by a dermatologist across five skin scales. We trained and evaluated machine learning models across 15 different configurations, including three neural network architectures and two loss functions.

**Results:**

The best‐performing model was an EfficientNet‐V2M architecture with a custom cross entropy loss. This model's mean test set accuracy across all labels was 85.41 ± 9.86 and its mean test set AUROC was 0.8306 ± 0.09599. An interesting trend emerged in which machine learning model performance was higher at the extremes of the scales, suggesting greater clinical ambiguity in the middle of the scales.

**Conclusions:**

Machine learning models are capable of predicting multiple skin characteristics simultaneously from color photographs of the face. In the future, similar models could assist non‐dermatologists in patient skin evaluation to enhance treatment planning.

## 1 Introduction

Evaluating skin characteristics such as Fitzpatrick skin type, hyperpigmentation, redness, and wrinkle severity is an important step in planning laser therapies [1], and demand for laser procedures is increasing. According to the ASDS Consumer Survey, in 2013, only 30% of consumers were considering getting a cosmetic procedure, but by 2023 this skyrocketed to 70% of consumers, with laser procedures as the most popular treatment under consideration [2].

In the United States, there is only one dermatologist for every 29 000 citizens [3]. To meet high patient demand, increasing numbers of nonphysicians are performing skin assessments and carrying out laser interventions, including nurses, aestheticians, cosmetologists, and unlicensed personnel at medical spas. Regulatory oversight remains poor, and in 73% of major US cities, medical spas now outnumber physician‐based cosmetic practices [4].

Unfortunately, medical spas have worse safety and outcomes than physician‐based practices [5, 6]. Incorrect evaluation of the skin can lead to serious patient harm, including pain, burns, skin discoloration, scarring, frostbite from the cooling system, infection, permanent disfigurement, and vision loss [5, 6, 7, 8, 9]. Prior work has shown that more complications occur when nonphysicians perform dermatologic procedures, and the most common reason for complications is improper technique by the nonphysician [6]. Dermatologists are able to understand the complex nuances of the skin and its physiology to apply the correct laser at the correct settings for each patient. This type of personalized medicine, with specific combinations of lasers and specific settings for each patient, is not possible at medical spas with non‐physician providers using lasers based on broad protocols which produce subpar results at best or complications at worst. A method of applying personalized medicine to lasers and cosmetic injections is needed.

Recent advances in artificial intelligence (AI) have led to impressive performance in computer vision tasks, including image classification, object detection, and segmentation [10, 11]. These advancements are based on a specific type of AI called machine learning, in which computers learn from data without being explicitly programmed. Machine learning has the potential to make dermatologist‐level expertise in skin analysis more accessible. In related work, Chang et al. trained neural network models on a small skin spectra dataset of approximately 200 images to classify Fitzpatrick skin type, reporting 81%–96% accuracy overall [12]. Saiwaeo et al. explored AI‐based classification of skin into normal, oily, and dry categories [13]. Groh et al. and Bencevic et al. uncovered bias in skin disease classification and segmentation models based on Fitzpatrick skin type [14, 15]. However, to the best of our knowledge there has been no prior work investigating whether machine learning models can evaluate multiple diverse skin characteristics simultaneously.

In this paper, we develop a new SkinAnalysis dataset comprising 3662 images, and use it to train and evaluate multilabel classification machine learning models that simultaneously predict Fitzpatrick skin type, hyperpigmentation, redness, and wrinkle severity.

## 2 Materials and Methods

### 2.1 Image Curation for the Novel SkinAnalysis Dataset

Machine learning models are only as good as the data on which they are trained. Unfortunately, we were not able to identify any image datasets annotated with Fitzpatrick skin type, hyperpigmentation, redness, and wrinkle severity. Thus, in order to train models on multiple skin characteristics, we developed a new dataset, the SkinAnalysis dataset, consisting of 3662 images (2928 train, 363 validation, and 371 test).

We used Internet images as the basis for this dataset due to the richness of facial photographs that are publicly available. Furthermore, although collecting a dataset in a controlled clinic setting might lead to higher model performance, this approach would also severely limit the model's generalization ability when deployed, as the model would not be exposed to sufficient variation in lighting, clothing, background, pose, and facial expression. We deliberately sought to create a dataset that would be diverse in both human and background characteristics, to facilitate model robustness. We included high‐quality face photos representing diverse skin tones, races, ethnicities, genders, ages, lighting, background, and clothing styles.

**FIGURE 1:**
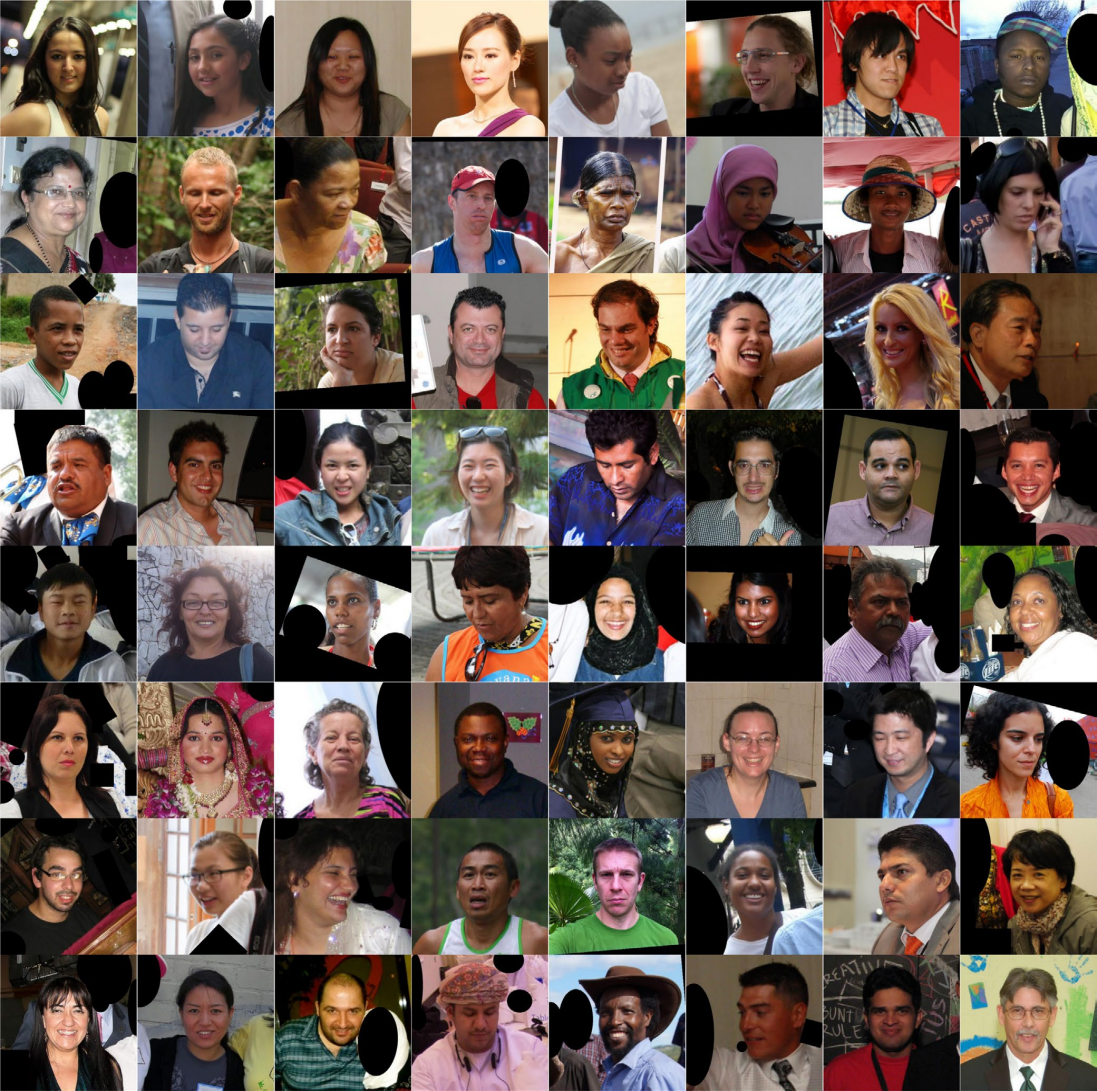
A random sample of 64 images from the SkinAnalysis dataset. Individuals of different genders, ethnicities, and races are included. Black circles and polygons are used to remove secondary subjects.

Images were sourced from FairFace [16], Openverse [17], and Wikipedia. All images went through an initial manual review by [R. Draelos] and/or a remote worker to ensure they were color images, depicting an adult, high enough resolution for all face details to be clearly visible, and taken at an angle that showed at least part of each eye. Images were excluded if they were black and white, low resolution, blurry, poorly lit such that face details were obscured, or in profile. Images were also excluded if they depicted a child, or if any part of the subject's face was blocked by another person or object. FairFace in particular included numerous images containing more than one person. For these images, we manually obscured any secondary people in the photographs using black polygons, so that only one person was the subject of the photograph; predicting the skin characteristics of multiple different individuals in the same picture was outside the scope of this project. When necessary, we also cropped images to center on the primary subject's face. Figure 1 depicts a selection of randomly‐chosen images from the final SkinAnalysis dataset.

### 2.2 Image Licenses

The FairFace dataset was released under a CC BY 4.0 license, and only includes images with either an “Attribution” or “Share Alike” Creative Commons license. All images sourced from Openverse or Wikipedia were public domain or licensed under CC BY or CC BY‐SA. We excluded images with NC (NonCommercial) licenses.

### 2.3 Image Annotation and Selection of Dermatologic Scales

[R. Draelos], a double board‐certified dermatologist and American Society for Dermatologic Surgery (ASDS) Fellowship Trained Cosmetic and Laser Surgeon, labeled all of the images with Fitzpatrick skin type (range 1–6), Kesty Pigmentation (range 0–3), Kesty Redness (range 0–4), Glogau Wrinkle Scale (range 1–4), and Fitzpatrick wrinkle severity (range 1–9) using LabelBox annotation software. See Table 1 for a data dictionary of the scales used (Table 1).

**TABLE 1:** Data dictionary of scales and definitions used in the artificial intelligence model.

Label (scale element)	Description
Fitzpatrick skin type 1	Always burns, never tans (palest; freckles); very light or white, “Celtic” type
Fitzpatrick skin type 2	Usually burns, tans minimally (light colored but darker than pale); light or light‐skinned European
Fitzpatrick skin type 3	Sometimes mild burn, tans uniformly (golden honey or olive); light intermediate, or dark‐skinned European
Fitzpatrick skin type 4	Burns minimally, always tans well (moderate brown); dark intermediate or “olive skin”
Fitzpatrick skin type 5	Very rarely burns, tans very easily (dark brown); dark or “brown” type
Fitzpatrick skin type 6	Never burns (deeply pigmented dark brown to darkest brown); very dark or “black” type
Kesty hyperpigmentation 0	No Pigmentation aside from base skin color
Kesty hyperpigmentation 1	Mild: Mild brown spots/patch/plaque covering 1%–25% of face (aside from base skin color)
Kesty hyperpigmentation 2	Moderate: Moderate brown with 25%–50% face surface area covered with abnormal hyperpigmentation
Kesty hyperpigmentation 3	Severe: > 50% of face surface area covered with additional hyperpigmentation above base skin color
Kesty redness 0	Clear skin with no signs of redness
Kesty redness 1	Almost Clear: Some mild or slight redness covering less than 10% of face surface area. Redness is almost imperceptible
Kesty redness 2	Mild: Mild redness covering 10%–25% of face surface area. Somewhat noticeable redness but cosmetically acceptable.
Kesty redness 3	Moderate: moderate redness covering 25%–50% of face surface area. Definitively cosmetically noticeable.
Kesty redness 4	Severe: Severe redness that covers > 50% of face surface area. Redness distracts from facial features
Glogau wrinkle scale 1	No wrinkles', early photo‐aging, mild pigment changes, no “age spots”
Glogau wrinkle scale 2	Wrinkles in motion, patient age 30's to 40's, early to moderate photo‐aging, appearance of lines only when face moves, early age “brown spots”, skin pores more prominent, early changes in skin texture
Glogau wrinkle scale 3	Wrinkles at rest, patient age 50s and older, advanced photoaging, prominent brown pigmentation, visible brown “age spots”, prominent small blood vessels, wrinkles now present with face at rest
Glogau wrinkle scale 4	Only wrinkles, patient age 60s or 70s, severe photoaging, wrinkles everywhere at rest or moving, yellow‐gray skin color, prior skin cancers, pre‐cancerous skin changes (actinic keratosis)
Fitzpatrick wrinkle severity 1	Mild: almost no fine textural changes with subtly accentuated skin lines
Fitzpatrick wrinkle severity 2	Mild: minimal fine textural changes with subtly accentuated skin lines
Fitzpatrick wrinkle severity 3	Mild: some fine textural changes with subtly accentuated skin lines
Fitzpatrick wrinkle severity 4	Moderate: minimal but distinct papular elastosis and dyschromia
Fitzpatrick wrinkle severity 5	Moderate: some but distinct papular elastosis and dyschromia
Fitzpatrick wrinkle severity 6	Moderate: noticeable distinct papular elastosis and dyschromia
Fitzpatrick wrinkle severity 7	Severe: some multipapular and confluent elastosis approaching or consistent with cutis rhomboidalis
Fitzpatrick wrinkle severity 8	Severe: distinct multipapular and confluent elastosis approaching or consistent with cutis rhomboidalis
Fitzpatrick wrinkle severity 9	Severe: severe multipapular and confluent elastosis approaching or consistent with cutis rhomboidalis

**FIGURE 2:**
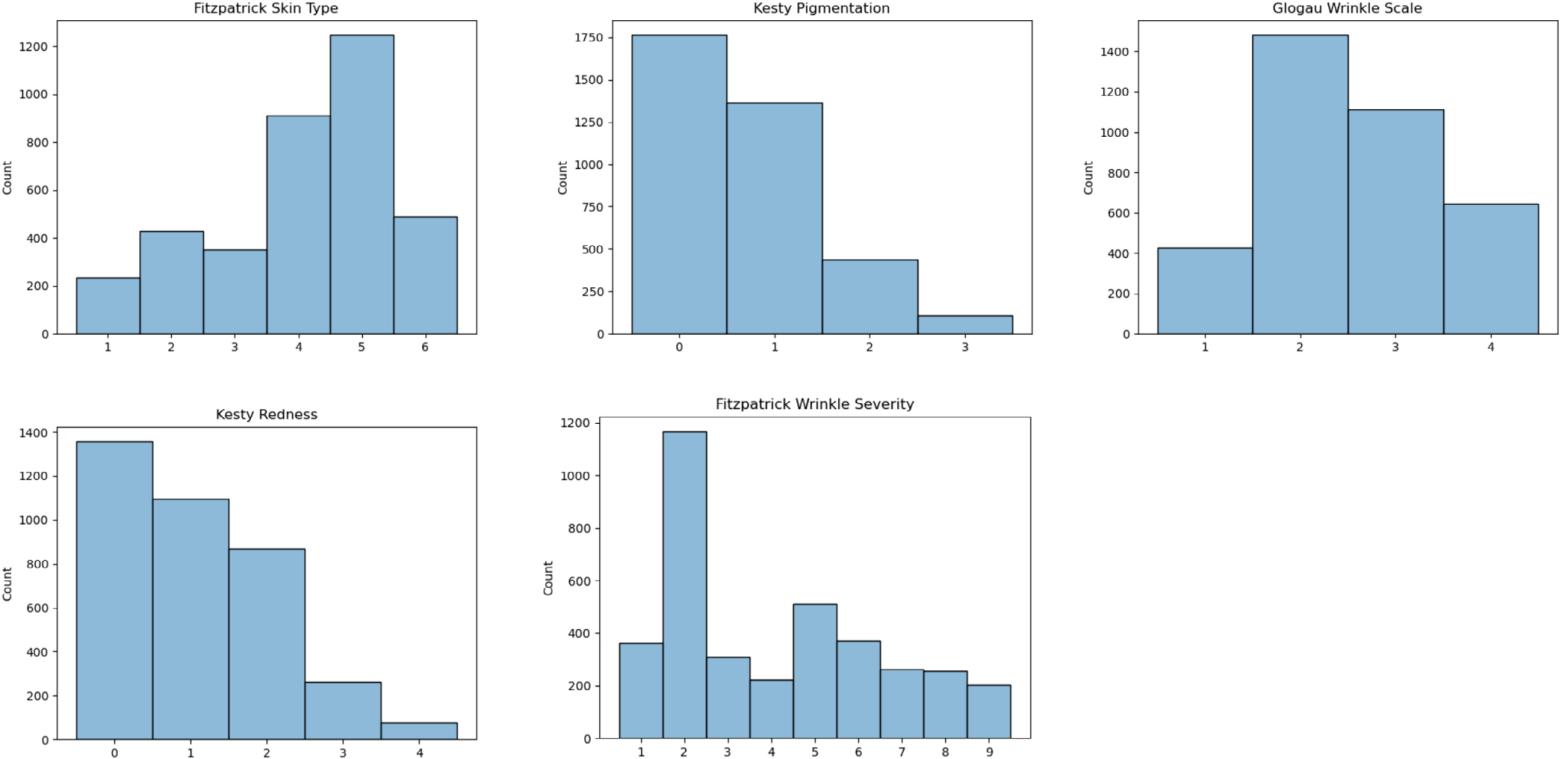
Histograms of the distribution of ground‐truth labels by skin scale. Although we specifically searched for images representing high redness and high pigmentation, these images were not prevalent in Internet databases and they are thus the smallest categories.

Figure 2 depicts histograms of the different scales across the entire dataset. Of note, in spite of conducting specific searches related to redness and hyperpigmentation, very few images ended up with the highest redness or hyperpigmentation scores. This suggests that individuals with severe facial redness or hyperpigmentation are underrepresented in online databases. However, even in these smaller categories, there are still on the order of 100 images.

### 2.4 Deep Learning Neural Network Architectures

We compared three established machine learning architectures for image classification: VGG‐16 [18], ResNet‐50 [19], and EfficientNet [20]. All models were pretrained on ImageNet [21], as pretraining on natural images has been shown to improve performance on medical imaging tasks [22]. The final fully connected layer of each model was replaced with a randomly initialized fully connected layer that predicted 28 outputs, one output for each value of each skin scale considered. All models were implemented in PyTorch.

### 2.5 Multiple Instance Learning

The standard preprocessing steps associated with each of the aforementioned architectures included a resampling step to a fixed input size. However, some of the images in the dataset were higher resolution than this fixed input size. We hypothesized that details contained in the higher resolution version of the images could be useful for prediction. We therefore explored a multiple instance learning approach [23] in which the model was applied to each of the four quadrants of the image separately, and a maximum taken over each output to produce the prediction for the whole image.

### 2.6 Data Augmentation

Data augmentation has been shown to improve classification performance [24]. We applied data augmentation to the training set, including random horizontal and vertical translations and flips, and random rotations in increments of 90°. We did not apply any data augmentation techniques that involved resampling, warping, or interpolation, as we did not want to distort image details. We also did not use any data augmentation techniques that would affect the color of the images.

### 2.7 Loss Functions

We compared three different loss functions: multilabel cross entropy, multiclass per‐scale cross entropy, and an ordinal regression loss.

#### 2.7.1 Multilabel Cross Entropy

This is a standard multilabel classification cross entropy loss with a sigmoid function applied to each output logit to convert it to a probability independently. This loss function does not prevent the network from predicting high probabilities for more than one element of the same scale, enabling the network to manifest more uncertainty. For example, the network could output high probabilities for both Glogau Wrinkle Scale = 3 and Glogau Wrinkle Scale = 4.

#### 2.7.2 Multiclass Per‐Scale Cross Entropy (SkinCELoss)

This is a more principled loss function that better aligns with the way skin scales actually work. In this loss function, the output vector of length M is considered by its scale subdivisions: m=0,…,5 for Fitzpatrick skin type, m=6,…,9 for Kesty Hyperigmentation, m=10,…,14 for Kesty Redness, m=15,…,18 for Glogau Wrinkle Scale, and m=19,…,27 for Fitzpatrick wrinkle severity. For each subdivision, we calculate a multiclass cross entropy loss:

$$\mathrm{CE}\left(y,\hat{y}\right)=-\frac{1}{C}\sum_{i=1}^{C}\left[y_{i}\log{\hat{y}}_{i}+\left(1-y_{i}\right)\log\left(1-{\hat{y}}_{i}\right)\right]$$

where, C refers to the total number of elements for that skin scale, and $y_{i}$ is a ground truth label for scale element i. The predicted probability ${\hat{y}}_{i}$ is calculated using the softmax function applied to the subset of C output neurons for that skin scale. The softmax makes each scale element mutually exclusive to other scale elements. As an example, for Fitzpatrick skin type, in this loss formulation increasing the probability of skin type = 3 means one or more other Fitzpatrick skin type probabilities need to decrease correspondingly.

The overall loss is a sum of the multiclass cross entropy losses for each skin scale. In the Tables, we refer to this overall loss as “SkinCELoss.” It prevents the network from predicting high probabilities for more than one element of any individual scale—i.e., for a particular image, the network cannot predict high probability for both Glogau Wrinkle Scale = 3 and Glogau Wrinkle Scale = 4 simultaneously; it must choose one.

#### 2.7.3 Ordinal Regression Loss

Each of the skin scales is ordered, so we also explored the NNRank method for ordinal regression with neural networks [25]. Unfortunately, this approach did not converge.

### 2.8 Training

Models were fine‐tuned on the SkinAnalysis dataset using an NVIDIA Titan RTX GPU with 24 GiB of memory, with early stopping on the validation set. Hyperparameter details are provided in the Appendix A: Tables A1 and A2.

### 2.9 Performance

We report accuracy and area under the receiver operating characteristic (AUROC). Accuracy can be inflated when labels are not balanced (e.g., in a case with 99% negative labels, a model that always outputs “no” is 99% accurate). We therefore additionally report AUROC, as AUROC does not suffer from the same artificial performance inflation issue. AUROC ranges from 0.5 (random classifier) to 1.0 (perfect classifier).

## 3 Results and Discussion

We observed multiple trends in machine learning model performance (Table 2). First, across configurations, the more modern EfficientNet architecture outperformed the older VGG‐16 and ResNet‐50 architectures. Second, data augmentation led to higher performance across 4 of 6 comparisons. Interestingly, the multiple instance learning approach led to consistently worse performance, with the “no MIL” approach always outperforming the “MIL max” approach. This suggests that the network benefits from processing the entire face all at once, possibly learning relationships between different parts of the face, rather than processing four quadrants of the face separately and only joining predictions at the end as seen in the MIL max approach.

**TABLE 2:** Validation set performance of all machine learning model configurations. The mean ± standard deviation of accuracy and AUROC across all labels is shown.

Architecture	MIL	Data Aug	Loss	Accuracy	AUROC
VGG‐16	No MIL	F	BCE	83.86 ± 11.6	0.7545 ± 0.1122
ResNet‐50	No MIL	F	BCE	84.13 ± 11.37	0.767 ± 0.1001
EfficientNet‐V2M	No MIL	F	BCE	84.21 ± 10.78	0.7976 ± 0.1064
VGG‐16	No MIL	T	BCE	84.08 ± 11.27	0.7853 ± 0.1026
ResNet‐50	No MIL	T	BCE	84.27 ± 11.18	0.7693 ± 0.1078
EfficientNet‐V2M	No MIL	T	BCE	84.3 ± 10.8	0.7943 ± 0.0992
VGG‐16	MIL max	F	BCE	83.32 ± 12.52	0.7309 ± 0.1096
ResNet‐50	MIL max	F	BCE	82.95 ± 11.62	0.738 ± 0.1049
EfficientNet‐V2M	MIL max	F	BCE	83.67 ± 11.63	0.7632 ± 0.1114
VGG‐16	MIL max	T	BCE	83.98 ± 11.43	0.7466 ± 0.1025
ResNet‐50	MIL max	T	BCE	82.05 ± 13.2	0.7062 ± 0.1139
EfficientNet‐V2M	MIL max	T	BCE	84.18 ± 11.18	0.7918 ± 0.105
VGG‐16	No MIL	T	SkinCELoss	83.91 ± 11.27	0.7841 ± 0.0974
ResNet‐50	No MIL	T	SkinCELoss	84 ± 11.5	0.7854 ± 0.1036
EfficientNet‐V2M	No MIL	T	SkinCELoss	**85.02 ± 10.47**	**0.8191 ± 0.0973**

*Note:* “Model” indicates the neural network architecture. “MIL” indicates whether multiple instance learning was used (MIL max) or not (no MIL). “Data Aug” indicates whether data augmentation was used (T) or not (F). Loss is either BCE (for multilabel cross entropy) or SkinCELoss (for multiclass per‐scale cross entropy). The best performance is indicated in bold font.

Overall, the best‐performing model was an EfficientNet‐V2M architecture with data augmentation, no multiple instance learning, and the SkinCELoss, a configuration we refer to as SkinScanNet (last row of Table 2). This model achieved a mean validation set accuracy of 85.02 ± 10.47, and a mean validation set AUROC of 0.8191 ± 0.0973. For detailed validation set performance of each model on a per‐label basis, see Appendix A: Tables A1 and A2.

This best‐performing model was applied to the test set (Table 3). The test set was only used once, at the conclusion of the study. The best model's mean test set accuracy was 85.41 ± 9.86 and its mean test set AUROC was 0.8306 ± 0.09599. This is similar to the validation set performance, indicating that the model generalizes well to unseen data.

**TABLE 3:** Test set performance of the final, best model, SkinScanNet, (EfficientNet‐V2M, no MIL, with data augmentation and the SkinCELoss).

Label (scale element)	Accuracy	AUROC
Fitzpatrick skin type 1	94.88	0.9644
Fitzpatrick skin type 2	89.22	0.8708
Fitzpatrick skin type 3	90.3	0.849
Fitzpatrick skin type 4	78.44	0.8074
Fitzpatrick skin type 5	78.98	0.8503
Fitzpatrick skin type 6	93.8	0.9579
Kesty hyperpigmentation 0	73.58	0.8069
Kesty hyperpigmentation 1	69	0.7121
Kesty hyperpigmentation 2	85.18	0.799
Kesty hyperpigmentation 3	97.04	0.9414
Kesty redness 0	68.19	0.7337
Kesty redness 1	73.32	0.6074
Kesty redness 2	76.01	0.6878
Kesty redness 3	92.18	0.7973
Kesty redness 4	97.84	0.9835
Glogau wrinkle scale 1	89.76	0.8896
Glogau wrinkle scale 2	70.89	0.7955
Glogau wrinkle scale 3	74.39	0.8124
Glogau wrinkle scale 4	93.26	0.9444
Fitzpatrick wrinkle severity 1	90.84	0.893
Fitzpatrick wrinkle severity 2	71.43	0.7813
Fitzpatrick wrinkle severity 3	91.11	0.7225
Fitzpatrick wrinkle severity 4	94.88	0.7472
Fitzpatrick wrinkle severity 5	83.02	0.7529
Fitzpatrick wrinkle severity 6	88.68	0.8063
Fitzpatrick wrinkle severity 7	93.8	0.8533
Fitzpatrick wrinkle severity 8	94.07	0.903
Fitzpatrick wrinkle severity 9	97.3	0.9871
Mean	85.41	0.8306
Standard deviation	9.86	0.09599

**FIGURE 3:**
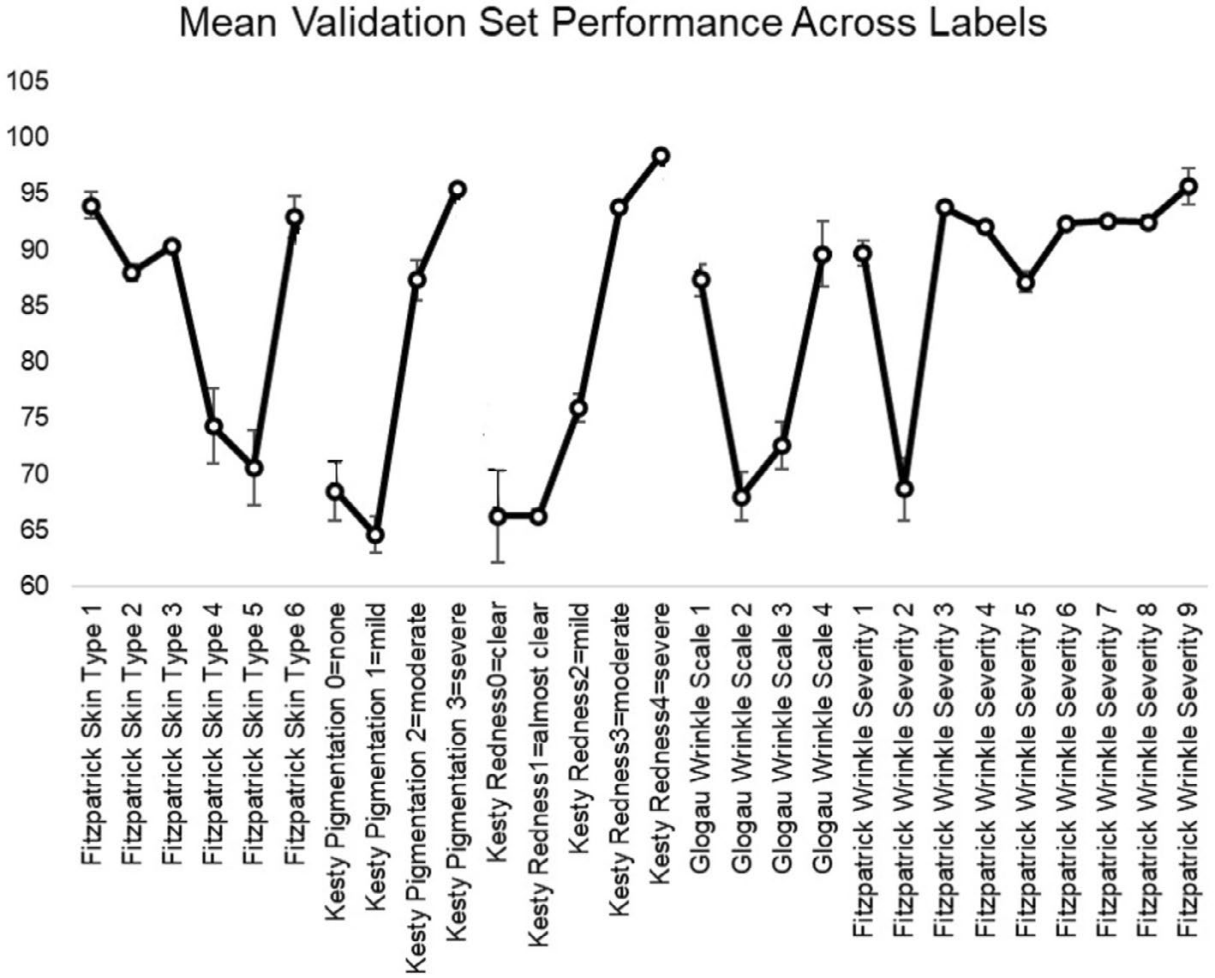
Mean validation set performance across each label to summarize the per‐label performance across 15 different model architectures and configurations considered. Performance at the extremes of each scale is higher than the performance in the middle of each scale.

**FIGURE 4:**
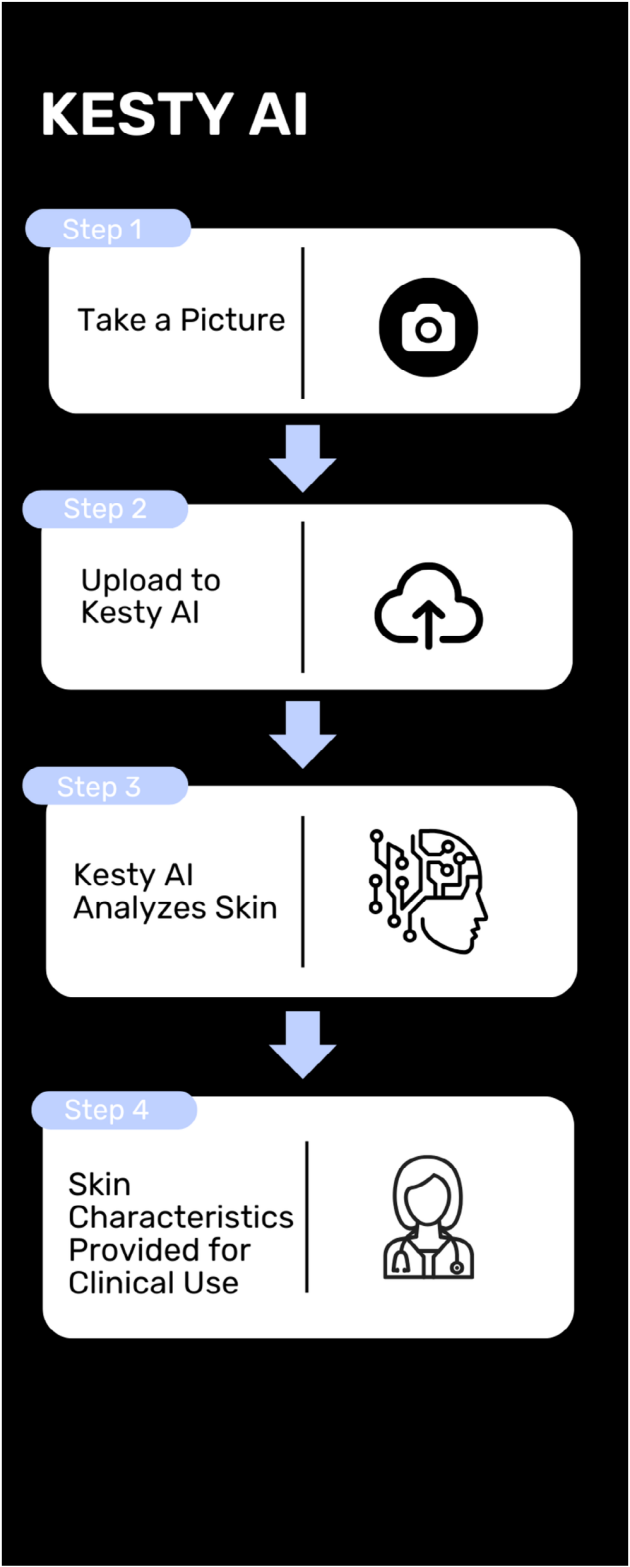
Flow chart of the process of using this artificial intelligence model to improve Dermatology clinical care.

Examining the per‐label (scale element) performance in Table 3 also illustrates an interesting trend: the performance is generally higher at the extremes of each scale, and lower in the middle. We plotted the mean validation set performance per scale element across all model configurations in order to better visualize this trend (Figure 3). Overall, the lower performance in the middle of each scale suggests that there is more clinical ambiguity in the middle, as it is more challenging for the model to distinguish intermediate levels of the scale. We believe this trend is a reflection of higher difficulty in the scale middles, rather than a simple indicator of number of training examples, because the performance does not directly track with number of examples. Comparing Figures 2 and 3 shows that sometimes performance is higher when number of examples is lower (e.g., Kesty Hyperigmentation = 3) and other times performance is lower when the number of examples is higher (e.g., Glogau Wrinkle Scale = 2). The consistent trend across all model configurations explored is for higher performance at the scale extremes (Figure 4).

### 3.1 Limitations

Our study has a few limitations. The SkinAnalysis dataset contains 3662 images, which is larger than the datasets of Chang et al. (200 images) [12], Saiwaeo et al. (329 images) [13], and Bencevic et al. (1758 images) [15], but smaller than that dataset of Groh et al. (16 577 images) [14]. Our labels were obtained through only one dermatologist, rather than a consensus of dermatologists, primarily due to the time consuming and expensive nature of dermatologist‐level expertise in data labeling. Across the 3662 images and five scales, [R. Draelos Dermatologist] produced 18 310 labels. In a future study it would be interesting to obtain labels on the SkinAnalysis dataset from other dermatologists, and evaluate inter‐dermatologist rating consistency as well as exploring how using a consensus label affects machine learning model performance. It would also be informative to collect labels on the SkinAnalysis dataset from non‐dermatologist physicians and non‐physician providers, and to compare machine learning model performance with the performance of non‐dermatologist practitioners. To be useful, a model does not have to outperform dermatologists—rather, it simply has to offer a higher level of expertise than other professionals who may be making assessments of skin characteristics during their careers.

Our study did calculate performance of the best model on a held‐out test set, but it did not calculate performance on an external test set collected in a different manner, as we were not able to identify any external datasets labeled with all the skin scales of interest^1^. Since SkinAnalysis was constructed from publicly available Internet images, it was also not clear how to manually construct a “separate” dataset from other Internet‐scraped images that would be sufficiently different from SkinAnalysis to be considered “external.” Prospective validation of the model on real‐time clinical cases is outside the scope of this preliminary work.

## 4 Conclusions

**FIGURE 5:**
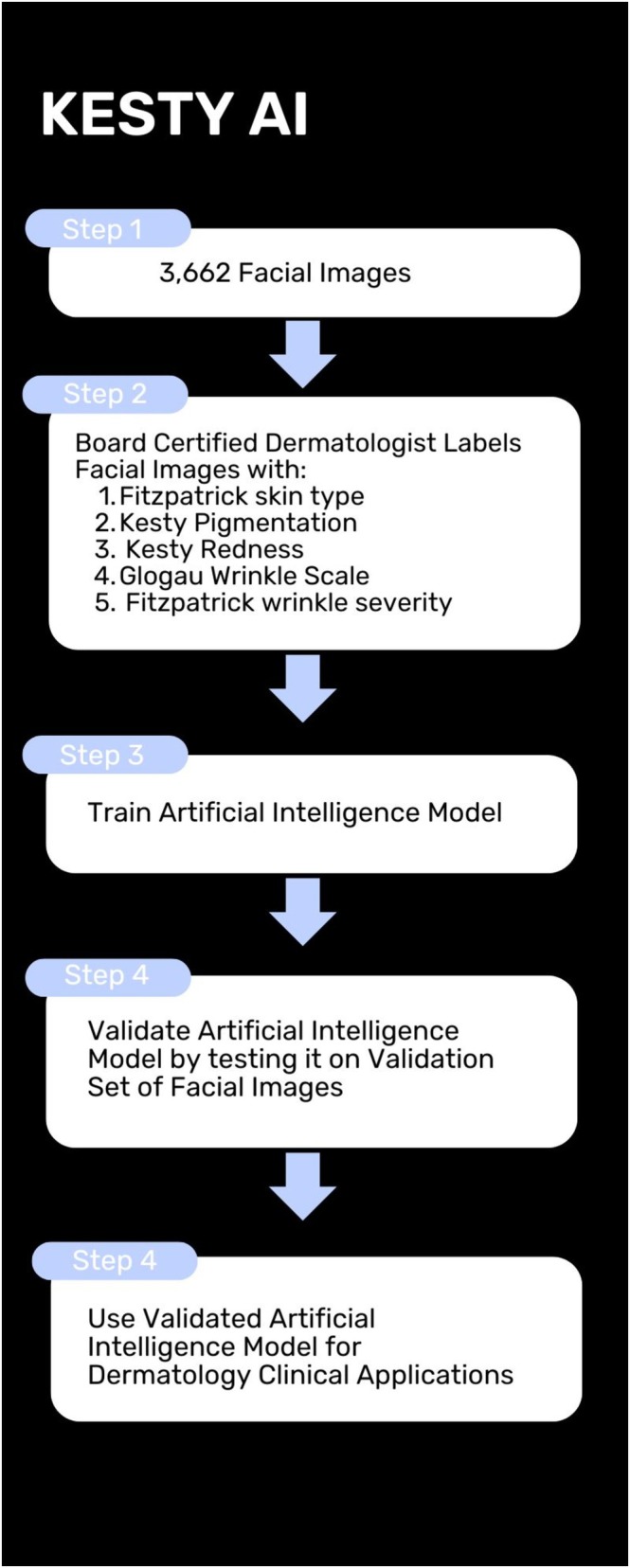
Graphical abstract of Kesty AI.

Overall, this is the first study to develop a machine learning model that predicts Fitzpatrick skin type, hyperpigmentation, redness, and wrinkle severity simultaneously from color photographs of the face (Figure 5). The model achieves extremely high performance on some scale elements, with accuracy > 90 and AUROC > 0.90, and achieves promising performance overall, with mean accuracy > 80 and mean AUROC > 0.80 across all scale elements. Strengths of our study include the diversity of images, representing individuals from all over the world and in a wide variety of settings, and the range of machine learning approaches we explore, including three architectures and two loss functions. It is our hope that this study will lead to safer and more effective treatment planning, by contributing to the development of future machine learning‐based tools that can augment the performance of non‐dermatologists who are already making skin assessments as part of their interactions with patients. Future work includes building on this model to output a personalized treatment plan for patients including potential laser wavelengths, laser settings, and cosmetic injection plans.

## Author Contributions

R.L.D. curated the photographs of the SkinAnalysis dataset, designed, trained, and evaluated all machine learning models, analyzed the results, and wrote the manuscript. K.R.K. and C.E.K. conceived the study, developed the Kesty Pigmentation and Kesty Redness scales, labeled the SkinAnalysis photographs, revised the manuscript, and funded the study. All authors have reviewed and approved the article for submission.

## Ethics Statement

This study leveraged only Creative Commons and public domain photographs freely and publicly available on the Internet. No patient data or protected health information was used at any point during the study.

## Conflicts of Interest

R.L.D. is the Founder and Principal Consultant of Vismedica AI, a healthcare AI consulting firm, and the Founder and CEO of Cydoc, an AI electronic health record startup. K.R.K. is the Founder of Kesty AI, which funded this work.

## Data Availability

The data that support the findings of this study are available on request from the corresponding author. The data are not publicly available due to privacy or ethical restrictions.

## Endnotes

^1^
We did initially consider calculating our model's performance on the Fitzpatrick 17 k dataset, but on further investigation, this dataset was designed for solving a different problem, namely to analyze bias in skin disease classification by skin type. This dataset is not of human faces, but instead consists of close‐up dermatology atlas images of diseased skin.

## Appendix A

**TABLE A1:** Validation set accuracy for all machine learning models considered. “Model” indicates the architecture.

Configuration															
Model	VGG‐16	ResNet‐50	EfficientNet‐V2M	VGG‐16	ResNet‐50	EfficientNet‐V2M	VGG‐16	ResNet‐50	EfficientNet‐V2M	VGG‐16	ResNet‐50	EfficientNet‐V2M	VGG‐16	ResNet‐50	EfficientNet‐V2M
Multiple instance learning (MIL)	No MIL	No MIL	No MIL	No MIL	No MIL	No MIL	MIL Max	MIL Max	MIL Max	MIL Max	MIL Max	MIL Max	No MIL	No MIL	No MIL
Data Aug	False	False	False	True	True	True	False	False	False	True	True	True	True	True	True
Loss function	BCE	BCE	BCE	BCE	BCE	BCE	BCE	BCE	BCE	BCE	BCE	BCE	SkinCELoss	SkinCELoss	SkinCELoss
Epoch	2	1	3	18	3	4	6	5	8	21	3	11	11	6	13
Accuracy															
fitz_skin_type_1	92.56	95.04	94.21	94.49	93.94	94.77	94.21	90.91	93.94	93.94	92.29	95.04	93.94	95.04	95.32
fitz_skin_type_2	88.15	87.88	86.78	87.88	88.15	87.6	87.88	89.26	87.33	88.43	86.78	88.98	88.71	87.88	87.88
fitz_skin_type_3	90.36	90.36	90.08	90.36	90.36	90.36	90.36	90.36	90.36	90.36	90.36	90.08	90.36	90.36	90.36
fitz_skin_type_4	73.83	74.1	75.21	74.1	76.31	78.79	73	73.55	75.76	76.03	63.64	74.66	74.93	73.83	76.58
fitz_skin_type_5	71.35	72.18	75.21	74.1	70.25	70.8	61.98	68.32	71.35	69.97	65.84	71.63	69.15	74.1	72.73
fitz_skin_type_6	94.21	93.94	93.39	91.74	94.21	92.84	92.56	90.36	93.94	92.29	88.98	93.66	90.63	95.87	95.04
kesty_pigmentation_0 = none	68.04	68.6	70.8	68.87	68.32	71.07	63.91	63.91	68.04	67.22	66.94	70.8	67.22	69.42	73.83
kesty_pigmentation_1 = mild	65.56	65.29	62.26	62.53	66.12	65.29	66.39	65.29	64.74	66.12	62.53	62.53	62.53	65.56	65.84
kesty_pigmentation_2 = moderate	85.4	87.6	85.95	87.33	89.53	82.92	88.43	88.71	87.88	89.81	87.88	86.23	87.05	88.98	85.67
kesty_pigmentation_3 = severe	95.59	95.59	94.77	95.32	95.59	95.59	95.59	95.04	95.59	95.59	95.59	95.32	95.04	95.59	95.32
kesty_redness_0 = clear	65.29	66.39	69.7	68.04	70.25	69.15	64.74	63.64	63.91	68.04	53.44	66.94	67.49	67.77	68.6
kesty_redness_1 = almost clear	66.39	66.39	67.22	65.56	66.39	65.56	66.67	65.56	65.84	66.67	65.29	66.94	66.39	66.39	66.67
kesty_redness_2 = mild	76.03	76.31	72.45	76.31	76.86	74.66	76.86	77.13	76.03	77.13	77.13	75.48	76.31	74.38	75.76
kesty_redness_3 = moderate	93.94	93.94	93.94	94.21	93.11	93.11	93.94	93.66	93.94	93.94	93.66	93.66	93.94	93.94	93.94
kesty_redness_4 = severe	98.35	98.35	98.35	98.9	98.35	98.07	98.35	98.07	98.07	98.35	98.35	98.07	98.62	98.35	98.35
glogau_wrinkle_scale_1	88.98	88.15	86.5	88.71	87.88	87.05	88.98	87.88	84.57	89.26	85.67	87.6	86.5	85.95	85.95
glogau_wrinkle_scale_2	66.39	66.67	69.7	66.94	66.39	68.32	67.49	66.39	69.15	67.77	65.29	72.73	69.7	65.56	71.63
glogau_wrinkle_scale_3	70.52	71.35	75.48	73.28	73.55	74.38	72.73	72.45	69.7	69.7	72.45	70.52	73	72.45	77.41
glogau_wrinkle_scale_4	90.08	90.91	93.94	90.36	89.53	91.74	88.15	83.2	90.91	88.43	83.47	91.46	90.08	89.81	92.29
fitz_wrinkle_severity_1	91.18	90.08	88.43	90.91	90.91	88.98	90.91	89.81	87.05	89.81	89.26	89.53	88.98	88.98	90.36
fitz_wrinkle_severity_2	68.87	70.52	71.35	70.52	67.49	71.9	62.26	68.04	67.22	66.67	65.56	69.7	71.63	66.39	72.18
fitz_wrinkle_severity_3	93.94	93.94	93.94	92.01	93.94	93.94	93.94	93.94	93.94	93.94	93.94	93.39	93.94	93.94	93.94
fitz_wrinkle_severity_4	92.01	92.01	92.01	92.01	92.01	92.01	92.01	92.01	92.01	92.01	92.01	92.01	92.01	92.01	92.01
fitz_wrinkle_severity_5	87.33	87.6	84.57	86.23	87.6	87.88	87.6	87.6	87.33	87.6	87.6	85.95	87.6	86.23	87.88
fitz_wrinkle_severity_6	92.56	92.56	90.91	92.56	92.56	92.29	92.56	92.56	92.56	92.56	92.56	92.29	92.56	92.56	91.46
fitz_wrinkle_severity_7	92.56	92.56	92.56	92.56	92.56	92.56	92.56	92.56	92.56	92.56	92.56	92.56	92.84	92.56	92.56
fitz_wrinkle_severity_8	92.84	92.56	92.01	91.46	93.11	92.56	92.84	91.74	92.84	91.74	92.56	92.56	91.74	92.84	93.39
fitz_wrinkle_severity_9	95.87	94.77	96.14	96.97	94.21	96.14	96.14	90.63	96.14	95.59	95.87	96.69	96.69	95.32	97.52
Mean	83.86	84.13	84.21	84.08	84.27	84.3	83.32	82.95	83.67	83.98	82.05	84.18	83.91	84	85.02
Standard deviation	11.6	11.37	10.78	11.27	11.18	10.8	12.52	11.62	11.63	11.43	13.2	11.18	11.27	11.5	10.47

*Note:* “Multiple Instance Learning” indicates whether a multiple instance learning strategy was used (MIL max) or not (no MIL). “Data Aug” indicates if data augmentation was used (TRUE) or not (FALSE). “Loss Function” indicates the loss function (BCE for multilabel cross entropy, or SkinCELoss). “Epoch” indicates the epoch selected by the early stopping process based on validation set performance.

**TABLE A2:** Validation set area under the receiver operating characteristic (AUROC) for all machine learning models considered. See description of Table A1 for row label explanations.

Configuration															
Model	VGG‐16	ResNet‐50	EfficientNet‐V2M	VGG‐16	ResNet‐50	EfficientNet‐V2M	VGG‐16	ResNet‐50	EfficientNet‐V2M	VGG‐16	ResNet‐50	EfficientNet‐V2M	VGG‐16	ResNet‐50	EfficientNet‐V2M
Multiple instance learning (MIL)	no MIL	no MIL	no MIL	no MIL	no MIL	no MIL	MIL max	MIL max	MIL max	MIL max	MIL max	MIL max	no MIL	no MIL	no MIL
Data Aug	False	False	False	True	True	True	False	False	False	True	True	True	True	True	True
Loss function	BCE	BCE	BCE	BCE	BCE	BCE	BCE	BCE	BCE	BCE	BCE	BCE	SkinCELoss	SkinCELoss	SkinCELoss
Epoch	2	1	3	18	3	4	6	5	8	21	3	11	11	6	13
AUROC															
fitz_skin_type_1	0.901	0.8935	0.9411	0.9346	0.936	0.9424	0.8755	0.8734	0.9584	0.8819	0.8656	0.9477	0.9298	0.911	0.9609
fitz_skin_type_2	0.8105	0.877	0.8653	0.8527	0.8575	0.8601	0.7922	0.8372	0.8331	0.7786	0.8116	0.8512	0.852	0.8656	0.888
fitz_skin_type_3	0.66	0.644	0.7025	0.6497	0.6637	0.7118	0.5641	0.6049	0.6041	0.6253	0.5626	0.685	0.6773	0.7199	0.7691
fitz_skin_type_4	0.7373	0.7381	0.7905	0.7509	0.7231	0.792	0.6938	0.6966	0.7402	0.7333	0.6667	0.7535	0.7675	0.7547	0.8014
fitz_skin_type_5	0.7546	0.7645	0.8202	0.7884	0.7639	0.7972	0.6656	0.7226	0.7501	0.7268	0.6625	0.784	0.7604	0.801	0.8316
fitz_skin_type_6	0.965	0.9706	0.9761	0.9572	0.9614	0.9709	0.9249	0.9282	0.9659	0.9191	0.8624	0.947	0.9393	0.9632	0.9672
kesty_pigmentation_0 = none	0.7624	0.7695	0.7826	0.7565	0.753	0.7798	0.7201	0.7315	0.7574	0.7476	0.7348	0.791	0.7608	0.7552	0.8136
kesty_pigmentation_1 = mild	0.6272	0.6155	0.6496	0.6206	0.6005	0.6539	0.6119	0.6484	0.653	0.6304	0.5415	0.6716	0.6413	0.6188	0.681
kesty_pigmentation_2 = moderate	0.6937	0.7248	0.7212	0.7276	0.75	0.7572	0.6524	0.724	0.7196	0.7211	0.7227	0.7564	0.6992	0.754	0.7963
kesty_pigmentation_3 = severe	0.8698	0.8642	0.8327	0.8201	0.8691	0.8655	0.7513	0.8453	0.8923	0.8222	0.8249	0.9332	0.9033	0.906	0.9321
kesty_redness_0 = clear	0.6632	0.7002	0.7398	0.714	0.7473	0.732	0.6433	0.694	0.6664	0.6793	0.6419	0.71	0.7379	0.6914	0.7421
kesty_redness_1 = almost clear	0.5655	0.6199	0.541	0.6341	0.5919	0.6082	0.5548	0.5405	0.5493	0.5784	0.5307	0.5777	0.6047	0.5727	0.6068
kesty_redness_2 = mild	0.5864	0.6595	0.6489	0.6746	0.6518	0.6349	0.5505	0.5948	0.6295	0.591	0.5743	0.6389	0.6689	0.6056	0.6616
kesty_redness_3 = moderate	0.7922	0.8738	0.8694	0.8444	0.8548	0.8443	0.7721	0.7998	0.8188	0.7973	0.7882	0.871	0.8256	0.8804	0.8644
kesty_redness_4 = severe	0.7908	0.8021	0.8492	0.8735	0.7843	0.817	0.7316	0.7932	0.8352	0.8133	0.7484	0.866	0.8721	0.8394	0.8847
glogau_wrinkle_scale_1	0.7783	0.742	0.8286	0.8553	0.8279	0.8301	0.8289	0.7442	0.7929	0.8082	0.7519	0.8442	0.8067	0.8282	0.8443
glogau_wrinkle_scale_2	0.74	0.7465	0.7787	0.771	0.7547	0.7969	0.7441	0.7205	0.7557	0.7273	0.7099	0.7942	0.7819	0.7559	0.7966
glogau_wrinkle_scale_3	0.7197	0.7403	0.7931	0.755	0.704	0.7843	0.7262	0.697	0.7048	0.6889	0.6827	0.7466	0.7789	0.7444	0.8047
glogau_wrinkle_scale_4	0.9295	0.9143	0.9675	0.9433	0.9176	0.9533	0.9012	0.9033	0.9127	0.9109	0.873	0.9442	0.9294	0.9225	0.9559
fitz_wrinkle_severity_1	0.7929	0.7777	0.842	0.8533	0.8268	0.839	0.8151	0.7539	0.7789	0.7874	0.7632	0.8466	0.8116	0.8404	0.8722
fitz_wrinkle_severity_2	0.7477	0.7633	0.7868	0.7705	0.7592	0.7951	0.7051	0.7196	0.7483	0.729	0.7075	0.7823	0.774	0.7547	0.786
fitz_wrinkle_severity_3	0.6293	0.6604	0.6873	0.6793	0.7017	0.6801	0.6436	0.6434	0.6745	0.6354	0.5499	0.6856	0.6925	0.725	0.7166
fitz_wrinkle_severity_4	0.6077	0.6669	0.7146	0.6953	0.6489	0.7321	0.7069	0.5825	0.7326	0.6847	0.5957	0.7467	0.7562	0.7157	0.7612
fitz_wrinkle_severity_5	0.6923	0.7037	0.7669	0.724	0.6514	0.7282	0.7218	0.6751	0.6925	0.648	0.615	0.6811	0.7265	0.6882	0.7656
fitz_wrinkle_severity_6	0.6426	0.6586	0.6985	0.6544	0.5989	0.6641	0.6178	0.6531	0.6046	0.641	0.5874	0.6699	0.6272	0.7108	0.6916
fitz_wrinkle_severity_7	0.8118	0.7722	0.8451	0.814	0.8382	0.8078	0.7278	0.753	0.7688	0.7642	0.6531	0.7896	0.8007	0.8438	0.8481
fitz_wrinkle_severity_8	0.8907	0.8854	0.9137	0.9108	0.8939	0.8926	0.881	0.8553	0.8941	0.8733	0.8008	0.9004	0.9042	0.8823	0.9197
fitz_wrinkle_severity_9	0.9647	0.9269	0.9792	0.9633	0.9104	0.9707	0.9422	0.9291	0.9346	0.9602	0.9456	0.9538	0.9258	0.9412	0.9718
Mean	0.7545	0.767	0.7976	0.7853	0.7693	0.7943	0.7309	0.738	0.7632	0.7466	0.7062	0.7918	0.7841	0.7854	0.8191
Standard deviation	0.1122	0.1001	0.1064	0.1026	0.1078	0.0992	0.1096	0.1049	0.1114	0.1025	0.1139	0.105	0.0974	0.1036	0.0973

Hyperparameter Details

All models trained with a BCE loss used the following hyperparameters:

- Learning rate = 0.001
- Weight decay = 1 × 10^−7^
- Maximum number of epochs = 150
- Patience (for early stopping) = 15
- Batch size = 4

All models trained with a SkinCELoss used the same hyperparameters, except that the learning rate was set lower, at 1 × 10^−5^, because when a learning rate of 0.001 was used, the models did not converge.
